# Endothelial Dysfunction in Psoriasis: A Comparative Analysis of Cutaneous, Inflammatory, and Metabolic Correlates

**DOI:** 10.3390/ijms27219675

**Published:** 2026-10-29

**Authors:** Tzeortziana Gkornik, Ana Olivia Toma, Codrina Mihaela Levai, Alexandru Caraba, Claudia Borza

**Affiliations:** 1 Doctoral School, Faculty of Medicine, “Victor Babes” University of Medicine and Pharmacy Timisoara, 300041 Timisoara, Romania; tzeortziana.gkornik@umft.ro (T.G.); codrinalevai@umft.ro (C.M.L.); 2 Department of Dermatology, “Victor Babes” University of Medicine and Pharmacy Timisoara, Eftimie Murgu Square 2, 300041 Timisoara, Romania; 3 Center for the Morphologic Study of the Skin (MORPHODERM), “Victor Babes” University of Medicine and Pharmacy Timisoara, 300041 Timisoara, Romania; 4 Research Center for Medical Communication, “Victor Babes” University of Medicine and Pharmacy Timisoara, 300041 Timisoara, Romania; 5 Department of Internal Medicine, Diabetes and Systemic Rheumatology, “Victor Babes” University of Medicine and Pharmacy Timisoara, 300041 Timisoara, Romania; caraba.alexandru@umft.ro; 6 Department of Functional Sciences, Discipline of Pathophysiology, “Victor Babes” University of Medicine and Pharmacy Timisoara, 300041 Timisoara, Romania; claudia.borza@umft.ro; 7 Centre of Cognitive Research in Pathological Neuro-Psychiatry NEUROPSY-COG, “Victor Babes” University of Medicine and Pharmacy Timisoara, 300041 Timisoara, Romania; 8 Centre for Translational Research and Systems, “Victor Babes” University of Medicine and Pharmacy Timisoara, Eftimie Murgu Square No. 2, 300041 Timisoara, Romania

**Keywords:** psoriasis, endothelial dysfunction, flow-mediated dilation, insulin resistance, tumor necrosis factor-α, cardiovascular risk, HOMA-IR

## Abstract

Psoriasis is associated with an elevated risk of cardiovascular disease, in which endothelial dysfunction, systemic inflammation, and insulin resistance are thought to interact. This study assessed endothelial function in 60 patients with psoriasis and 30 age- and sex-matched healthy controls, and examined its relationships with disease severity (PASI), insulin resistance (HOMA-IR), and tumor necrosis factor-α (TNF-α). Endothelial function was measured as brachial-artery flow-mediated dilation (FMD). Compared with controls, patients showed significantly reduced FMD together with higher TNF-α, HOMA-IR, renal resistive index, albumin-to-creatinine ratio, body mass index, and total cholesterol, and lower high density lipoprotein (HDL)-cholesterol (all *p* ≤ 0.002). Within the patient group, PASI correlated strongly and positively with TNF-α and HOMA-IR, and inversely with FMD, while both TNF-α and the renal resistive index correlated inversely with FMD (all *p* < 0.001). In separate multivariable regression models, a model containing TNF-α and HOMA-IR explained a slightly larger proportion of variance in FMD (R^2^ = 0.72) than a model containing PASI and HOMA-IR (R^2^ = 0.70); in both, HOMA-IR was the most consistently associated predictor, while PASI did not reach significance (standardized β = −0.18, *p* = 0.08). When all three predictors were entered simultaneously, strong collinearity between PASI and TNF-α (variance inflation factors 4.8 and 4.5, respectively) precluded reliable separation of their effects, and only HOMA-IR and TNF-α retained significance. These associations persisted after adjustment for age, sex, BMI, systolic blood pressure, and smoking status.These findings indicate that insulin resistance is the strongest independently associated factor of endothelial dysfunction in psoriasis, with systemic inflammation contributing additional explanatory value; however, the strong overlap between disease severity and circulating TNF-α prevents definitive separation of their individual effects, and formal mediation analyses in larger cohorts are warranted.

## 1. Introduction

Psoriasis represents a chronic inflammatory skin disease defined clinically by relapsing scaly plaques, affecting approximately 2% of the population in Europe and North America, with reported prevalence ranging from under 1% to 8.5% across adult populations worldwide [1,2]. What began as a diagnosis defined by skin and joint findings is now understood far more broadly [3]. The umbrella term “psoriatic disease” has gained acceptance precisely because the disorder extends beyond these two compartments and includes a spectrum of cardiometabolic comorbidities [4,5]. Rates of both cardiovascular complications and serious cardiac events run higher in this population than in the general population [6,7,8]. Metabolic risk factors are more prevalent among these patients, yet they do not account for the full excess risk, as the disease itself appears to exert an independent effect [1,9]. Psoriasis carries a 57% higher cardiovascular mortality even after adjustment for conventional risk factors [10,11].

Inflammation and endothelial dysfunction reinforce one another, and this reciprocal relationship is thought to accelerate atherosclerosis, produce subclinical organ damage, and eventually manifest as overt cardiovascular disease [1]. In psoriasis, much of the inflammatory input derives from Th1 and Th17 activation, which elevates circulating levels of mediators including TNF-α, IL-6 and IL-1β [11,12,13]. Exposed to this milieu, endothelial cells shift toward an activated state, upregulating chemotactic, pro-atherogenic and adhesion molecules, with arterial inflammation and direct injury from circulating cytokines as downstream consequences [1]. Among these, E-selectin and ICAM-1 govern the stepwise recruitment of leukocytes across the endothelium, a process through which immune cells reach the dermis and contribute to atherosclerotic plaque formation [14]. This sequence has been formalized as the “psoriatic march,” in which systemic inflammation drives insulin resistance, insulin resistance impairs endothelial function, and atherosclerosis follows [15,16].

Insulin resistance therefore occupies a central position in this pathway, and it is frequently present in psoriasis. Beyond its metabolic role, insulin functions as a vasoactive hormone, stimulating endothelial nitric oxide synthesis and exerting anti-inflammatory effects on the vessel wall; impaired insulin signaling therefore has direct consequences for vasomotor tone and endothelial homeostasis [1]. More broadly, insulin resistance has been proposed as a unifying mechanism through which chronic inflammatory conditions confer excess cardiovascular risk, also promoting endothelial injury [17]. Direct tissue evidence exists as well; Akt phosphorylation, the readout of intact insulin signaling, is diminished in the dermal endothelium of psoriatic patients relative to healthy skin. The same deficit appears in skin that is clinically uninvolved, and in individuals whose glycated hemoglobin is entirely normal, raising the possibility that the vasculature becomes insulin resistant locally well before any metabolic abnormality is detectable systemically [14].

Endothelial dysfunction is characterized by impaired endothelium-dependent vasodilation, attributable primarily to reduced nitric oxide bioavailability, and represents an early, well-established event in the initiation and progression of atherosclerosis [1]. Inflammation-driven reductions in nitric oxide bioavailability appear before cardiovascular disease becomes clinically evident [11]. Brachial artery flow-mediated dilation (FMD) is the most extensively studied non-invasive method for evaluating endothelial function and carries prognostic weight for subsequent cardiovascular events, particularly among higher-risk groups [1], although its sensitivity to operator technique, patient preparation, and inter-laboratory protocol differences remain a recognized limitation [1]. A meta-analysis of psoriatic patients and matched controls found brachial-artery FMD significantly reduced and carotid intima-media thickness significantly increased relative to controls, differences that persisted even in participants free of conventional atherosclerotic risk factors [2]. This impairment extends to smaller vessels, as direct measurement in the cutaneous microvasculature has revealed reduced nitric oxide-dependent vasodilation that tracked with the extent of skin involvement [11].

Whether cutaneous disease severity, systemic inflammation, or insulin resistance carries the largest independent association with endothelial damage in psoriasis remains insufficiently characterized. The present study was designed to address this question by measuring endothelial function in patients with psoriasis and age- and sex-matched healthy controls, and relating it to three potential contributors: skin disease severity (PASI), insulin resistance (HOMA-IR), and systemic inflammation (TNF-α). The principal aim was to determine which of these retains an independent association with impaired endothelial function after accounting for the others. We hypothesized that circulating TNF-α and insulin resistance would be more closely tied to endothelial dysfunction than skin disease severity alone, since PASI captures the extent of cutaneous involvement but may not directly reflect the systemic metabolic and inflammatory burden acting on the vessel wall.

## 2. Results

### 2.1. Comparison Between Patients and Controls

Patients with psoriasis (n = 60) were compared with age- and sex-matched healthy controls (n = 30). The two groups did not differ significantly in age (*p* = 0.751), sex distribution (*p* = 0.332), or smoking status (*p* = 0.937). Median disease duration was 15 years. The majority of patients had severe disease, with 48 (80.0%) having a PASI ≥ 20. Patients with psoriasis also had significantly higher systolic and diastolic blood pressure than controls, with 51 (85.0%) recording a study-visit blood pressure ≥140/90 mmHg. They also showed significantly higher levels of TNF-α, HOMA-IR, renal resistive index (RI), urinary albumin-to-creatinine ratio (ACR), body mass index (BMI), and total cholesterol, together with significantly lower flow-mediated dilation (FMD) and HDL-cholesterol (all *p* ≤ 0.002; Table 1).

### 2.2. Correlations Between Disease Severity, Inflammation, Insulin Resistance, and Endothelial Function

**Figure 1:**
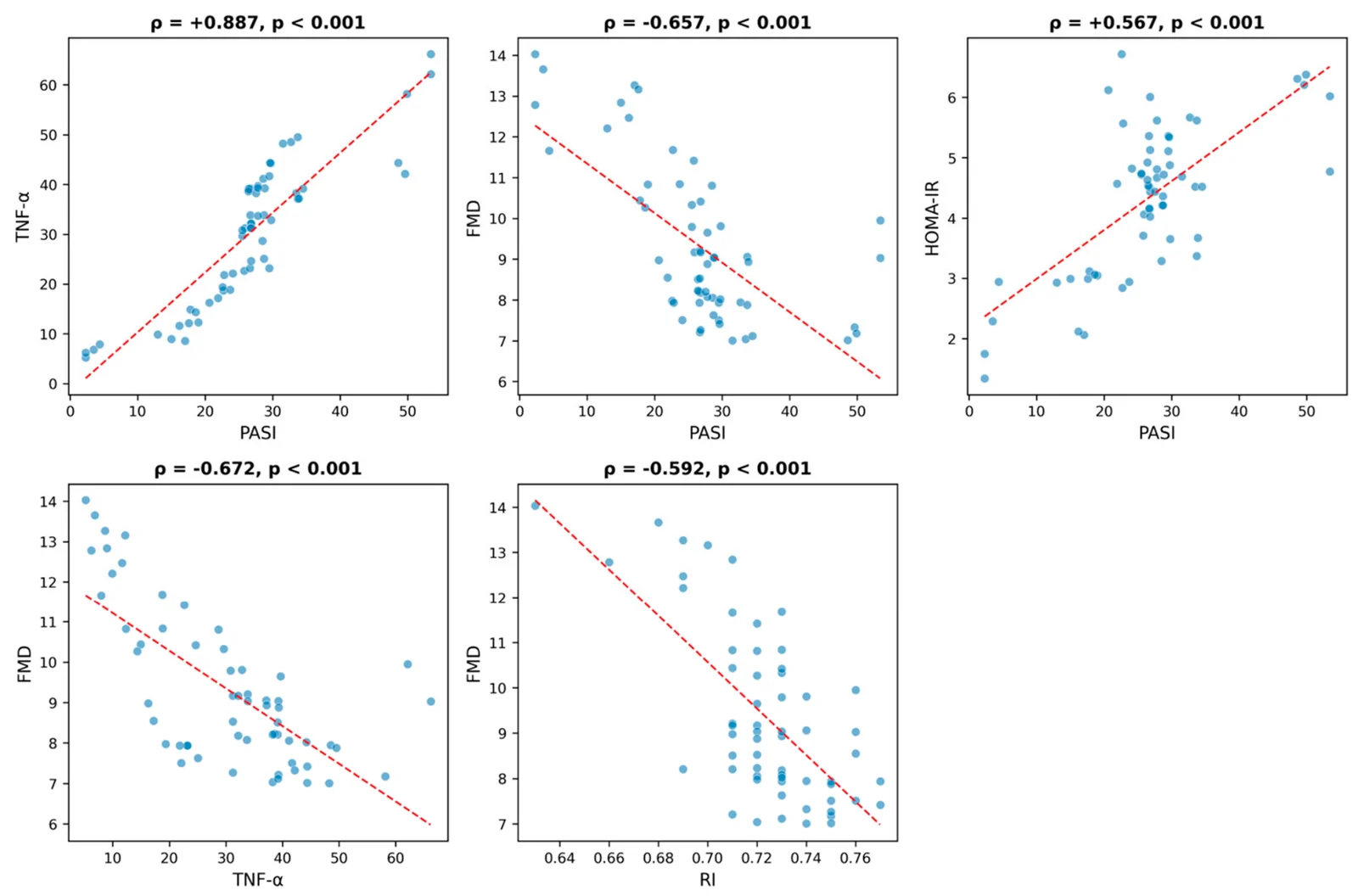
Scatter plots with linear regression lines for each analyzed pair of variables in the cutaneous psoriasis group. Spearman (ρ) coefficients are displayed above each panel.

In the psoriasis group, disease severity assessed by PASI showed a strong positive correlation with the pro-inflammatory cytokine TNF-α (ρ = +0.887, *p* < 0.001) and with insulin resistance quantified by HOMA-IR (ρ = +0.567, *p* < 0.001). PASI correlated inversely with endothelial function measured by flow-mediated dilation (FMD; ρ = −0.657, *p* < 0.001). TNF-α was likewise negatively associated with FMD (ρ = −0.672, *p* < 0.001), and the RI correlated inversely with FMD (ρ = −0.592, *p* < 0.001). All five associations were statistically highly significant (Table 2, Figure 1).

### 2.3. Distribution of Study Variables

The distribution of each variable was assessed using the Shapiro–Wilk test (Table S1). PASI, FMD, and RI deviated significantly from normality (*p* < 0.05), whereas TNF-α and HOMA-IR were normally distributed. Because at least one non-normally distributed variable was present in every analyzed pair, the Spearman rank correlation coefficient was adopted as the primary measure of association.

### 2.4. Factors Independently Associated with Endothelial Function

To identify the factors independently associated with endothelial function, multivariable linear regression models were fitted with FMD as the dependent variable. Because PASI and TNF-α were strongly collinear (ρ = 0.89), three models were constructed to allow comparison rather than a single model from which one predictor was excluded a priori. Model 1 included PASI and HOMA-IR; Model 2 included TNF-α and HOMA-IR; Model 3 included all three predictors.

**Figure 2:**
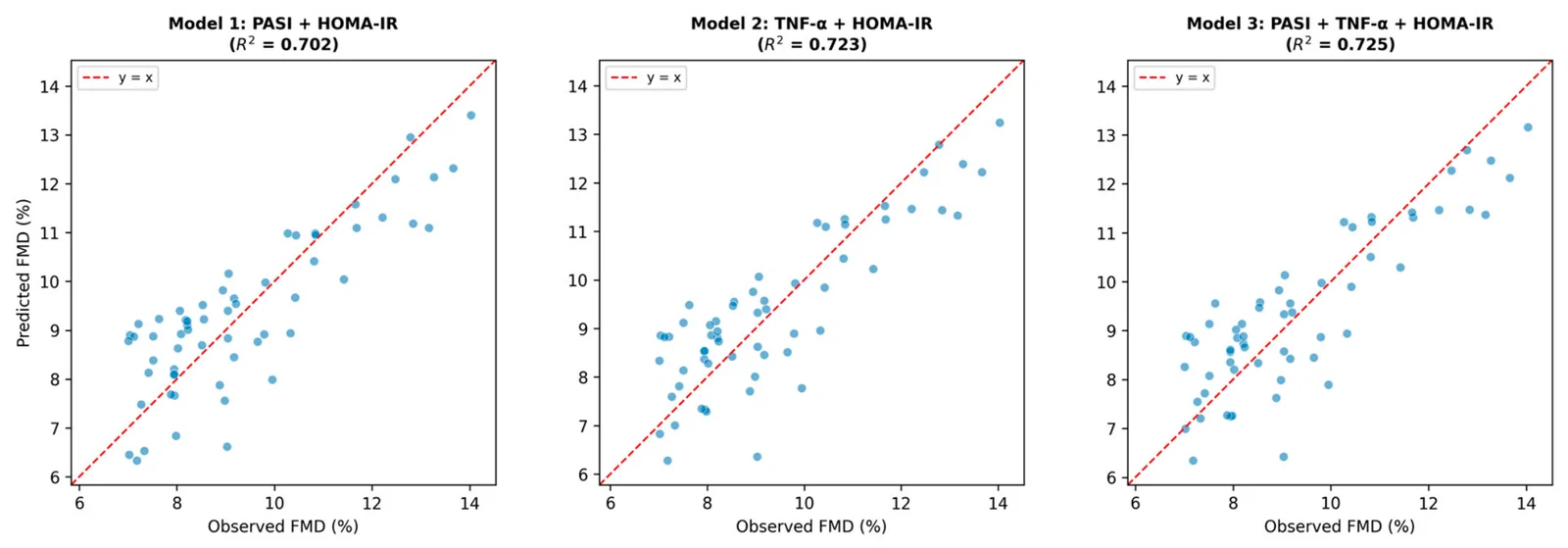
Observed versus predicted FMD values from three multivariable regression models. Dashed lines represent perfect agreement (y = x).

In Model 1, HOMA-IR was a strong independently associated factor (*p* < 0.001), whereas PASI did not reach statistical significance (*p* = 0.083); the model explained 70.2% of the variance in FMD. In Model 2, both TNF-α (*p* = 0.007) and HOMA-IR (*p* < 0.001) were significant, and the model explained a larger proportion of variance. Model 3, which included all three predictors, TNF-α and HOMA-IR remained significant, while PASI was not significant and its coefficient reversed sign; the inflated variance of the PASI and TNF-α coefficients in this model limits the interpretability of their individual contributions. Model fit did not improve meaningfully over Model 2. Across all three models, insulin resistance was consistently the strongest independently associated factor of impaired endothelial function (Table 3, Figure 2). AIC values were 180.7 for Model 1, 176.1 for Model 2, and 177.9 for Model 3. The difference between Models 2 and 3 was 1.8 units; therefore, Model 2 is preferred as the more parsimonious specification.

### 2.5. Adjusted Models

Among the covariates, only systolic blood pressure was significantly associated with FMD (*p* < 0.001). HOMA-IR, TNF-α, and PASI were then each added separately to this base model. HOMA-IR retained a strong and significant association with FMD after full adjustment (Model B, *p* < 0.001; R^2^ = 0.740), as did TNF-α (Model C, *p* < 0.001; R^2^ = 0.676). PASI remained non-significant (Model D, *p* = 0.086; R^2^ = 0.600), consistent with the unadjusted analysis. Full model coefficients are reported in Supplementary Table S2.

## 3. Discussion

In this study, patients with psoriasis exhibited significantly impaired endothelial function compared with healthy controls, alongside higher levels of systemic inflammation, insulin resistance, and adiposity. Within the patient group, endothelial dysfunction correlated with skin disease severity (PASI), inflammation (TNF-α), and insulin resistance (HOMA-IR). In multivariable analysis, HOMA-IR was the strongest independently associated factor of FMD across all models tested, while TNF-α contributed additional explanatory value; PASI did not reach significance in any model specification.

These findings are consistent with the current understanding of the vascular biology of psoriasis. Systemic inflammation is thought to promote insulin resistance through cytokine-mediated interference with insulin signaling in endothelial cells, reducing nitric-oxide–dependent vasodilation and thereby inducing endothelial dysfunction [16,18,19]. TNF-α, a central cytokine in psoriasis [20], acts as a major insulin antagonist [21], providing a mechanistic link between the inflammatory and metabolic pathways observed in our data. Insulin resistance in psoriasis appears to reflect a true metabolic disturbance rather than an artifact of surrogate indices [22]. A prospective cohort of 405 patients and 134 age-, sex- and body mass index–matched controls demonstrated significantly elevated fasting insulin, C-peptide and HOMA-IR despite comparable glucose and glycated hemoglobin, indicating that the defect is frequently subclinical and escapes glucose-based screening [22]. Reduced insulin sensitivity in psoriasis has also been demonstrated using other methods: in a hyperinsulinemic euglycemic clamp study, normal glucose-tolerant patients with moderate-to-severe psoriasis showed significantly lower insulin sensitivity than matched controls, although HOMA-IR did not differ between the groups [23]. The link between disease severity and insulin resistance is further supported across independent psoriasis cohorts, in which HOMA-IR was higher in patients than controls and rose with PASI [24,25], though this association is not universal, weakening once metabolic syndrome is taken into account [26].

The regression analysis suggests that insulin resistance was the most strongly and consistently associated factor across all model specifications, while the contributions of disease severity and systemic inflammation could not be reliably separated due to their strong collinearity. PASI did not reach significance even in Model 1, where TNF-α was absent. Replacing PASI with TNF-α (Model 2) resulted in a modestly higher R^2^ and lower AIC, which may suggest that circulating TNF-α reflects systemic inflammation more directly than PASI; however, given the small sample size and the strong correlation between PASI and TNF-α, this interpretation remains speculative and requires confirmation in larger studies. When all three predictors were entered together (Model 3), the strong collinearity between PASI and TNF-α inflated coefficient variance, PASI reversed sign, and only TNF-α and HOMA-IR retained significance; however, this pattern does not constitute formal evidence that inflammation mediates the PASI–FMD association. An alternative explanation, that PASI exerts a direct effect the model lacked precision to detect, or that both variables index overlapping aspects of the same inflammatory process, cannot be excluded without dedicated mediation analysis in a larger sample. These findings were consistent in the adjusted analysis: when added separately to the base model, both HOMA-IR and TNF-α each retained a significant association with FMD after adjustment for age, sex, BMI, systolic blood pressure, and smoking status, while PASI did not; however, as HOMA-IR and TNF-α were not modelled together in the adjusted analysis, their associations cannot be considered mutually independent. In the adjusted models, VIF values for systolic blood pressure in Model B (4.20) and BMI in Models C and D (4.19 and 4.24) slightly exceeded the prespecified threshold of 4, which reflects the known correlation between adiposity and blood pressure rather than a structural modeling problem; given that all values remained well below 5 and the coefficients of interest were consistent across models, this is unlikely to affect the conclusions materially.

The molecular basis for the observed associations lies in the convergence of inflammatory and metabolic signaling on the endothelial nitric oxide pathway. The Th1/Th17-dominant cytokine milieu, driven primarily by TNF-α, IL-17, and IL-23, extends beyond the skin to exert systemic vascular effects [27,28]. TNF-α activates NF-κB signaling in endothelial cells, upregulating adhesion molecules (ICAM-1, VCAM-1, E-selectin) and promoting leukocyte recruitment to the vessel wall, while simultaneously suppressing endothelial nitric oxide synthase (eNOS) expression and activity [16,29,30]. In parallel, TNF-α disrupts insulin signaling through serine phosphorylation of insulin receptor substrate-1 (IRS-1), which inhibits the downstream PI3K/Akt pathway and further reduces eNOS-derived nitric oxide bioavailability [31,32]. This pathway creates a self-reinforcing loop in which inflammation and metabolic dysfunction jointly erode endothelial homeostasis. Skin endothelial cells may retain long-term transcriptional and epigenetic changes following psoriatic inflammation, potentially contributing to disease recurrence and the persistence of vascular dysfunction beyond clinical remission [33]. The psoriatic skin itself contributes to this systemic change, where activated dendritic cells and keratinocytes release TNF-α, IL-6, and IL-1β into the circulation, while the expanded body of inflamed skin functions as a diffuse endocrine organ amplifying systemic cytokine levels in proportion to disease extent [20,34], which may partly explain the strong PASI–TNF-α correlation observed in this cohort. Smoking may contribute to endothelial dysfunction in psoriasis through overlapping inflammatory mechanisms, with the activation of NF-κB signaling, increased reactive oxygen species generation, and stimulation of innate immune cells that perpetuate cytokine-driven inflammation; however, in this study, smoking prevalence did not differ significantly between groups [35]. Furthermore, in the predominantly obese patients of this cohort, visceral fat constitutes an independent source of TNF-α and IL-6 and contributes to insulin resistance through adipokine dysregulation (reduced adiponectin, elevated leptin and resistin), compounding the inflammatory drive originating from the skin. The result is a cumulative effect of cutaneous, adipose, and hepatic signals on the vascular endothelium, where reduced nitric oxide availability manifests clinically as impaired flow-mediated dilation.

Prior analyses have consistently demonstrated reduced FMD in psoriatic patients compared with healthy controls, leading to the view that disease severity itself contributes to vascular impairment and that more severely affected patients should be targeted for cardiovascular screening [36]. These findings, however, were based on group-level comparisons and did not test whether PASI predicts vascular impairment within the patient group once systemic inflammatory and metabolic factors are taken into account. The present analysis was designed to address that gap and our bivariate analysis confirmed a significant PASI–FMD correlation; PASI failed to reach significance even in Model 1, where TNF-α was absent and only HOMA-IR was adjusted for. This could suggest that previously reported PASI–FMD associations may have been partly or wholly confounded by covariant metabolic and inflammatory factors that were not accounted for. If endothelial dysfunction tracks with the systemic inflammatory and metabolic burden rather than with skin involvement, then a patient with modest skin disease but elevated TNF-α and insulin resistance may carry more vascular risk than one with widespread plaques but a quiescent systemic milieu. The biological distinction between cutaneous microvascular and systemic macrovascular endothelial dysfunction may also contribute to the weak independent association between PASI and brachial-artery FMD observed in this study.

Experimental evidence supports insulin resistance as a proximate contributor in this pathway: dermal endothelial cells in psoriatic skin show reduced Akt phosphorylation indicative of endothelial insulin resistance, detectable even in non-lesional skin of patients with normal glycated hemoglobin [14].

Our comparative findings are in agreement with prior studies that documented elevated TNF-α and HOMA-IR together with reduced brachial-artery FMD in psoriatic arthritis relative to controls. Insulin resistance correlated positively with TNF-α (r = 0.65) and inversely with brachial-artery FMD (r = −0.80), while disease activity was associated with both greater insulin resistance (r = 0.81) and poorer endothelial function (r = −0.84) [37]. A comparable pattern was reported in a case–control study of 60 patients with chronic plaque psoriasis and 30 matched controls, in which serum TNF-α was approximately threefold higher in patients than in controls and correlated positively with PASI (*p* < 0.001) [38]. Additionally, a systematic review concluded that patients with psoriasis and psoriatic arthritis show impaired endothelium-dependent vasodilation and increased arterial stiffness compared with the general population [39]. Disease severity itself has been linked to subclinical vascular disease, with carotid intima-media thickness increasing in parallel with PASI score [8]. In our cohort, endothelial dysfunction was not confined to the brachial artery: the renal resistive index was significantly higher in patients than in controls and correlated inversely with FMD.

Blockade of TNF-α has been linked to a more favorable cardiovascular risk profile [40,41], an effect attributed at least in part to improved endothelial cell behavior [1]. Whether this translates into measurable recovery of endothelial function is less certain. One report found that TNF-α inhibition enhanced resting endothelium-dependent vascular tone, gauged by low-flow-mediated constriction, without any accompanying change in brachial flow-mediated dilation [42], and early studies of FMD gave inconsistent results. At the level of clinical outcomes, however, the evidence is more consistent. More recent real-world data reinforce a cardiovascular benefit: in a large propensity-matched cohort of over 12,000 psoriasis patients, biologic therapy was associated with a substantially lower five-year risk of incident cardiovascular disease relative to oral anti-psoriatic treatment, with significant reductions for TNF-α, IL-17 and IL-23 inhibitors but not for IL-12/23 blockade [43]. A separate body of data likewise indicates that TNF-α inhibitor therapy may lower the risk of major adverse cardiac events relative to topical or phototherapy, possibly through plaque stabilization and improved endothelial function [44]. Consistent with this, psoriatic patients treated with methotrexate have shown lower insulin resistance and improved endothelial function than untreated patients [37].

The higher renal resistive index and urinary albumin-to-creatinine ratio, together with the inverse RI–FMD correlation within the patient group, suggest that endothelial impairment in psoriasis extends beyond the brachial artery to the renal microvasculature. Another study that assessed the renal artery resistive index reported significantly higher microalbuminuria in psoriatic patients, together with positive correlations between albuminuria and both PASI score and renal artery resistive index [45].

The markedly higher body mass index fits the well-recognized association between psoriasis and adiposity [46,47], and adipose tissue is itself a source of pro-inflammatory cytokines and a contributor to insulin resistance [46,48]. The less favorable lipid profile, with higher total cholesterol and lower HDL-cholesterol, is typical of the atherogenic dyslipidemia that often accompanies psoriasis and represents a further, independent layer of cardiovascular risk. The BMI difference between patients and controls was substantial and obesity independently impairs endothelial function and elevates insulin resistance [49]; the observed associations cannot therefore be attributed to psoriasis alone. However, in a sensitivity analysis, BMI was not independently significant and model fit did not improve, suggesting that the effect of adiposity on FMD was already captured by HOMA-IR. The significantly higher blood pressure in the patient group is consistent with the well-documented association between psoriasis and hypertension [50] and represents an additional contributor to the endothelial dysfunction observed.

This study has several limitations. Its cross-sectional design precludes causal inference, and the single-center setting with a modest sample size may limit generalizability and statistical power for subgroup effects. The strong collinearity between PASI and TNF-α prevented reliable separation of their individual contributions; the non-significance of PASI may reflect genuine mediation through inflammation, insufficient power, or overlap between the two measures, and cannot be resolved without formal mediation analysis in a larger sample. Insulin resistance was estimated using HOMA-IR, a fasting surrogate that primarily reflects hepatic sensitivity and may underestimate peripheral and vascular insulin resistance relative to reference clamp methods. Additionally, TNF-α was measured as a single fasting sample, and residual variability due to circadian fluctuation, subclinical infection, or psychological stress cannot be excluded. Blood pressure was recorded as a single study-visit measurement and cannot be equated with a formal arterial hypertension diagnosis. The BMI difference between groups reflects the association between psoriasis and adiposity; however, excess weight independently impairs endothelial function and cannot be fully disentangled from the effect of psoriasis itself, despite the sensitivity analysis suggesting that the adiposity effect was largely captured by HOMA-IR. Prior systemic or biologic treatment exposure and concomitant topical therapy were not systematically recorded and cannot be excluded as sources of residual confounding. Finally, the absence of longitudinal follow-up precludes conclusions about whether the observed associations translate into subsequent cardiovascular events or respond to treatment.

## 4. Materials and Methods

### 4.1. Study Design and Population

This study was conducted between 2019 and 2026. The study group comprised patients with psoriasis, and the control group comprised healthy volunteers. Controls were frequency-matched to patients by age (within 5-year bands) and sex to achieve comparable group-level distributions. The diagnosis of psoriasis was established clinically by a dermatologist and confirmed histopathologically where necessary. All participants provided written informed consent, and the study was approved by the Ethics Committee of the Railway Clinical Hospital Timisoara, Romania, with registration number 140/July 2019 and conducted in accordance with the principles of the Declaration of Helsinki [51].

### 4.2. Inclusion and Exclusion Criteria

Patients were eligible if they were aged 18–70 years and had a confirmed diagnosis of chronic plaque psoriasis. To avoid confounding of the metabolic and vascular measurements, patients were excluded if they had diabetes mellitus, established cardiovascular or cerebrovascular disease, chronic kidney disease, active infection, malignancy, or pregnancy, or if they were receiving systemic or biologic antipsoriatic therapy or any medication known to affect vascular function or glucose metabolism. Healthy controls had no history of psoriasis and were excluded if they had any inflammatory disorder, established cardiovascular or cerebrovascular disease, chronic kidney disease, or diabetes mellitus. Patients with concomitant psoriatic arthritis were excluded, as the additional systemic inflammatory changes and distinct cytokine profile associated with joint involvement could independently affect both endothelial function and insulin resistance, confounding the assessment of cutaneous disease severity as a predictor.

Recruitment was consecutive throughout the study period; the extended duration of enrollment reflects the combined effect of strict eligibility criteria, reduced patient flow during the COVID-19 pandemic, and the low prevalence of eligible patients at a single center. Approximately 130 patients with chronic plaque psoriasis were assessed for eligibility over the study period, of whom 60 were enrolled. The principal reasons for exclusion were concomitant diabetes or established cardiovascular disease.

### 4.3. Clinical Assessment and Disease Severity

Clinical assessment, laboratory sampling, and vascular measurements were all performed during the same study visit. In patients with psoriasis, disease severity was quantified using PASI. PASI was scored by a dermatologist before blood sampling and vascular assessment, with all three components completed within a single morning session (typically within 2–3 h). No minimum disease-stability interval was imposed as an inclusion criterion; however, patients were enrolled at routine scheduled visits rather than during acute flares requiring urgent care, and the cohort therefore predominantly reflects chronic stable plaque psoriasis. BMI was calculated as body weight in kilograms divided by height in metres squared. Blood pressure was recorded after the participant had rested for at least 10 min in a seated position.

### 4.4. Laboratory Measurements

Venous blood samples were drawn between 07:30 and 09:00 h after an overnight fast of at least 12 h. Participants were instructed to abstain from caffeine, alcohol, and vigorous physical exercise for at least 12 h before the visit. Samples were centrifuged at 3000 rpm for 10 min within 30 min of collection and stored at −80 °C until analysis. Patients with clinical signs of acute infection at the time of the study visit were rescheduled. Serum TNF-α was quantified by chemiluminescent immunoassay (QuantiGlo ELISA Kit, R&D Systems Inc., Minneapolis, MN, USA, CLIA-serum limit of detection 1.7 pg/mL) according to the manufacturer’s instructions. Fasting plasma glucose was measured by the enzymatic hexokinase method and serum insulin by electrochemiluminescence immunoassay (ECLIA, Roche/Hitachi Cobas analyzers, Hitachi High-Tech Corporation, Tokyo, Japan); insulin resistance was estimated using the homeostatic model assessment (HOMA-IR), calculated as fasting insulin (µIU/mL) × fasting glucose (mg/dL)/405. Total cholesterol and high-density lipoprotein cholesterol (HDL-c) were determined by standard enzymatic methods. The urinary albumin-to-creatinine ratio (ACR) was determined from spot urine samples.

### 4.5. Assessment of Endothelial Function

Endothelial function was assessed non-invasively by FMD of the brachial artery using a high-resolution ultrasound system (Siemens Acuson X300 Ultrasound System, with linear transducer of 10 MHz, Siemens, Munich, Germany). Measurements were performed with the participant resting in a supine position in a temperature-controlled room, after fasting and abstaining from caffeine. The brachial artery was imaged above the antecubital fossa and the baseline diameter recorded. A sphygmomanometer cuff was then inflated on the forearm to 50 mmHg above systolic pressure for 5 min; following deflation, the brachial artery diameter was measured again 60 s after cuff release. FMD was expressed as the percentage change in arterial diameter relative to baseline.

All measurements were performed by a single experienced vascular sonographer throughout the study. The operator was aware of participant group allocation, as blinding was not feasible given the visible nature of psoriatic skin lesions; however, the operator was blinded to all laboratory results at the time of the vascular assessment. Brachial artery diameters were measured on end-diastolic frames gated to the R-wave of a concurrent electrocardiographic recording, and each value represents the mean of three consecutive cardiac cycles. Intra-observer reproducibility was assessed by repeated measurements in 10 randomly selected participants on a separate occasion within 7 days; the coefficient of variation for baseline brachial artery diameter was 2.8% and for FMD was 8.4%.

### 4.6. Renal Doppler Assessment

RI was determined by Doppler ultrasonography of the intrarenal arteries and calculated as (peak systolic velocity—end-diastolic velocity)/peak systolic velocity.

### 4.7. Statistical Analysis

The normality of continuous variables was evaluated with the Shapiro–Wilk test. Differences between patients and controls were assessed using the Mann–Whitney U test, with values expressed as median [interquartile range]. Correlations between parameters within the patient group were assessed using the Spearman rank correlation coefficient (ρ), selected because PASI, FMD, and RI were not normally distributed and each analyzed pair contained at least one non-normal variable. Pearson correlation coefficients were computed in parallel as a sensitivity analysis and were consistent in direction and significance for all pairs; the Spearman coefficient was therefore adopted as the primary measure of association. A two-tailed *p*-value < 0.05 was considered statistically significant. Independently associated factors of endothelial function were assessed by multivariable linear regression with FMD as the dependent variable. Predictors with a variance inflation factor > 4 were considered collinear. Because PASI and TNF-α exceeded this threshold when entered together, three models were compared: Model 1 (PASI + HOMA-IR), Model 2 (TNF-α + HOMA-IR), and Model 3 (all three predictors). Variance inflation factors are reported for each model. Model comparison was further informed by the Akaike information criterion (AIC). Model fit was expressed as R^2^ and adjusted R^2^, and both unstandardized and standardized regression coefficients are reported. Regression diagnostics were examined for all three models. Normality of residuals was confirmed by the Shapiro–Wilk test (all *p* > 0.50), homoscedasticity was assessed by the Breusch–Pagan test (Model 1: BP = 5.97, *p* = 0.051; Model 2: BP = 5.95, *p* = 0.051; Model 3: BP = 6.84, *p* = 0.077); and independence of residuals was supported by Durbin–Watson statistics close to 2 (Model 1: 1.90; Model 2: 1.88; Model 3: 1.86). Influential observations were evaluated using Cook’s distance; no observation exceeded the conventional threshold of 1.0 (maximum Cook’s D = 0.37 in Model 2), and results were stable when the three observations with Cook’s D exceeding 4/n were excluded in a sensitivity analysis. To address potential cardiovascular confounding, a base model was prespecified on clinical grounds, including age, sex, BMI, systolic blood pressure, and smoking status. PASI, TNF-α, and HOMA-IR were then each added separately to this base model to assess whether their associations with FMD persisted after adjustment. Diagnostic plots for the preferred model (Model 2) are provided in Supplementary Figure S1. Statistical analyses were performed using Python version 3.11.

## 5. Conclusions

In patients with psoriasis, brachial-artery FMD was significantly reduced compared with controls; however, the marked differences in adiposity and blood pressure between groups mean that this finding cannot be attributed to psoriasis alone. Insulin resistance and systemic inflammation were more closely associated with endothelial impairment than cutaneous disease severity, which did not retain significance in any model after adjustment. Prospective studies with formal mediation analysis are needed to establish the direction and clinical relevance of these associations.

## Figures and Tables

**Table 1:** Comparison of clinical and biological parameters between patients with psoriasis and healthy controls. Values are median [interquartile range].

Parameter	Patients	Controls	*p*-Value
Age (years)	44.00 [37.00–51.00]	42.00 [38.25–47.75]	0.751
Male sex, *n* (%)	34 (56.7)	13 (43.3)	0.332
Smoking status, *n* (%)			
Current	17 (28.3)	10 (33.3)	0.937
Former	3 (5)	1 (3.3)	
Never	40 (66.7)	19 (63.3)	
Disease duration (years)	15 [10–18]		
PASI	26.8 [22.7–29.5]		
Mild (<10), *n* (%)	4 (6.7)		
Moderate (10–<20), *n* (%)	8 (13.3)		
Severe (≥20), *n* (%)	48 (80.0)		
TNF-α (pg/mL)	31.70 [18.81–39.25]	3.91 [2.65–6.23]	<0.001
HOMA-IR	4.52 [3.35–5.12]	1.38 [1.11–1.47]	<0.001
FMD (%)	8.96 [7.94–10.43]	13.23 [12.38–14.60]	<0.001
RI	0.72 [0.71–0.74]	0.63 [0.62–0.68]	<0.001
ACR (mg/g)	54.26 [43.09–67.99]	10.98 [7.56–24.96]	<0.001
BMI (kg/m^2^)	33.02 [31.13–34.60]	24.53 [22.82–28.41]	<0.001
Total cholesterol (mg/dL)	198.50 [180.00–203.50]	175.00 [160.50–197.25]	0.002
HDL-c (mg/dL)	50.00 [44.00–55.00]	62.50 [54.00–65.75]	<0.001
SBP (mmHg)	145 [140–150]	110 [105–130]	<0.001
DBP (mmHg)	100 [95–100]	75 [70–84]	<0.001

*p*-values from the Mann–Whitney U test, except for sex distribution and smoking status, which were compared using the chi-square test. For smoking status, current and former smokers were combined into a single “ever smoker” category for the purpose of group comparison. PASI-Psoriasis Area and Severity Index; TNF-α-tumor necrosis factor alpha; HOMA-IR-homeostatic model assessment of insulin resistance; FMD-flow-mediated dilation; RI-renal resistive index; ACR-albumin-to-creatinine ratio; BMI,-body mass index; HDL-c-high-density lipoprotein cholesterol; SBP-systolic blood pressure; DBP-diastolic blood pressure.

**Table 2:** Spearman rank correlations between clinical and biological parameters in the psoriasis group.

Parameter Pair	Spearman ρ	*p*-Value
PASI vs. TNF-α	+0.887	<0.001
PASI vs. FMD	−0.657	<0.001
PASI vs. HOMA-IR	+0.567	<0.001
TNF-α vs. FMD	−0.672	<0.001
RI vs. FMD	−0.592	<0.001

ρ, Spearman rank correlation coefficient.

**Table 3:** Multivariable linear regression models with FMD as the dependent variable.

Model	Predictor	β (Unstd.)	95% CI	Std. β	*p*-Value	VIF
Model 1 (R^2^ = 0.702, adj. R^2^ = 0.691)	PASI	−0.032	[−0.069, 0.004]	−0.177	0.083	1.92
	HOMA-IR	−1.097	[−1.410, −0.785]	−0.705	<0.001	1.92
Model 2 (R^2^ = 0.723, adj. R^2^ = 0.714)	TNF-α	−0.035	[−0.060, −0.010]	−0.263	0.007	1.81
	HOMA-IR	−1.014	[−1.306, −0.722]	−0.652	<0.001	1.81
Model 3 (R^2^ = 0.725, adj. R^2^ = 0.710)	PASI	+0.014	[−0.041, 0.070]	+0.079	0.607	4.77
	TNF-α	−0.043	[−0.083, −0.003]	−0.322	0.034	4.50
	HOMA-IR	−1.037	[−1.345, −0.729]	−0.667	<0.001	1.99

β, regression coefficient; 95% CI, 95% confidence interval; Std. β, standardized coefficient; VIF, variance inflation factor.

## Data Availability

Data are available on request from the corresponding author.

## Supplementary Materials

The following supporting information can be downloaded at: https://www.mdpi.com/article/10.3390/ijms27219675/s1.

## References

[1] Anyfanti P., Margouta A., Goulas K., Gavriilaki M., Lazaridou E., Patsatsi A., Gkaliagkousi E. (2022). Endothelial Dysfunction in Psoriasis: An Updated Review. Front. Med..

[2] Fang N., Jiang M., Fan Y. (2016). Association Between Psoriasis and Subclinical Atherosclerosis: A Meta-Analysis. Medicine.

[3] Billi A.C., Gudjonsson J.E., Voorhees J.J. (2019). Psoriasis: Past, Present, and Future. J. Investig. Dermatol..

[4] Larid G., Delwail A., Dalle T., Vasseur P., Silvain C., Jégou J.-F., Morel F., Lecron J.-C., Gervais E. (2022). Ex Vivo Cytokine Production in Psoriatic Disease: Towards Specific Signatures in Cutaneous Psoriasis and Peripheral Psoriatic Arthritis. Front. Immunol..

[5] Sobchak C., Eder L. (2017). Cardiometabolic Disorders in Psoriatic Disease. Curr. Rheumatol. Rep..

[6] Jindal S., Jindal N. (2018). Psoriasis and Cardiovascular Diseases: A Literature Review to Determine the Causal Relationship. Cureus.

[7] Ponikowska M., Hill L., Lee C.S., Barisone M., Ponikowski P., Aldossary H.M., Jędrzejczyk M., Kubielas G., Uchmanowicz I. (2026). Cardiovascular Disease and Psoriasis. Dermatol. Ther..

[8] Wu J.J., Kavanaugh A., Lebwohl M.G., Gniadecki R., Merola J.F. (2022). Psoriasis and Metabolic Syndrome: Implications for the Management and Treatment of Psoriasis. J. Eur. Acad. Dermatol. Venereol..

[9] Hajiebrahimi M., Song C., Hägg D., Andersson T.M.-L., Villacorta R., Linder M. (2020). The Occurrence of Metabolic Risk Factors Stratified by Psoriasis Severity: A Swedish Population-Based Matched Cohort Study. Clin. Epidemiol..

[10] Mehta N.N., Azfar R.S., Shin D.B., Neimann A.L., Troxel A.B., Gelfand J.M. (2010). Patients with Severe Psoriasis Are at Increased Risk of Cardiovascular Mortality: Cohort Study Using the General Practice Research Database. Eur. Heart J..

[11] Alba B.K., Greaney J.L., Ferguson S.B., Alexander L.M. (2018). Endothelial Function Is Impaired in the Cutaneous Microcirculation of Adults with Psoriasis through Reductions in Nitric Oxide-Dependent Vasodilation. Am. J. Physiol.-Heart Circ. Physiol..

[12] Diani M., Altomare G., Reali E. (2016). T Helper Cell Subsets in Clinical Manifestations of Psoriasis. J. Immunol. Res..

[13] Orzan O.A., Tutunaru C.V., Ianoși S.L. (2025). Understanding the Intricate Pathophysiology of Psoriasis and Related Skin Disorders. Int. J. Mol. Sci..

[14] Schlüter K., Diehl S., Lang V., Kaufmann R., Boehncke W., Bürger C. (2016). Insulin Resistance May Contribute to Upregulation of Adhesion Molecules on Endothelial Cells in Psoriatic Plaques. Acta Derm. Venereol..

[15] Boehncke W.-H., Boehncke S., Tobin A.-M., Kirby B. (2011). The ‘Psoriatic March’: A Concept of How Severe Psoriasis May Drive Cardiovascular Comorbidity: The ‘Psoriatic March’. Exp. Dermatol..

[16] Boehncke W.-H. (2018). Systemic Inflammation and Cardiovascular Comorbidity in Psoriasis Patients: Causes and Consequences. Front. Immunol..

[17] Haroon M., Gallagher P., Heffernan E., FitzGerald O. (2014). High Prevalence of Metabolic Syndrome and of Insulin Resistance in Psoriatic Arthritis Is Associated with the Severity of Underlying Disease. J. Rheumatol..

[18] Paneni F. (2015). Role of Oxidative Stress in Endothelial Insulin Resistance. World J. Diabetes.

[19] Venkatesan K., Paulsamy P., Alqahtani S., Asiri Y.I., Alqahtani T., Alqahtani A., Sivadasan D., Ahmed R., Elfadil H., Asseri K.A. (2025). An Update on the Role of Inflammation in Insulin Resistance and Islet β Cell Failure- A Crosstalk between Immune Cells, Adipose Tissue, Cytokines, and Oxidative Stress. Lett. Drug Des. Discov..

[20] Matei-Man A.-M., Vesa Ș.C., Pușcaș A.D., Orăsan M.S., Homorozeanu B., Candrea E., Mocan T. (2025). Assessment of TNF-α, IL-12/23, and IL-17 in Psoriasis: Only TNF-α Reflects Clinical Response After 12 Weeks of Biologic Treatment. Curr. Issues Mol. Biol..

[21] Hotamisligil G.S., Murray D.L., Choy L.N., Spiegelman B.M. (1994). Tumor Necrosis Factor Alpha Inhibits Signaling from the Insulin Receptor. Proc. Natl. Acad. Sci. USA.

[22] Cheung S., Wang H., Wu J., Zhang L., Ruan Y., Zhou J., Wang Y., Chen L., Pan M., Li X. (2026). Insulin Resistance in Psoriasis: Metabolic Recalibration and Therapeutic Prediction; a Prospective Cohort Study at a Tertiary Referral Centre. Br. J. Dermatol..

[23] Gyldenløve M., Storgaard H., Holst J.J., Vilsbøll T., Knop F.K., Skov L. (2015). Patients with Psoriasis Are Insulin Resistant. J. Am. Acad. Dermatol..

[24] Mohamad N., Elshimi E. (2020). Relation between Insulin Resistance and Severity of Psoriasis in Egyptian Patients. Egypt. J. Dermatol. Venerol..

[25] Wardhana M., Adiguna M.S., Nareswari P.A.D. (2022). Positive Correlation of Psoriasis Vulgaris Severity and HOMA-IR. Bali Dermatol. Venereol. Aesthetic J..

[26] Levakov O., Gajinov Z., Gajic B., Vujanovic L., Ivkov-Simic M., Golusin Z. (2022). Psoriatic Arthritis and Psoriasis Severity as Metabolic Syndrome and Insulin Resistance Predictors. Srp. Arh. Celok. Lek..

[27] Girolomoni G., Strohal R., Puig L., Bachelez H., Barker J., Boehncke W.H., Prinz J.C. (2017). The Role of IL-23 and the IL-23/TH 17 Immune Axis in the Pathogenesis and Treatment of Psoriasis. J. Eur. Acad. Dermatol. Venereol. JEADV.

[28] Li L., Liu J., Lu J., Wu J., Zhang X., Ma T., Wu X., Zhu Q., Chen Z., Tai Z. (2025). Interventions in Cytokine Signaling: Novel Horizons for Psoriasis Treatment. Front. Immunol..

[29] Min J.-K., Kim Y.-M., Kim S.W., Kwon M.-C., Kong Y.-Y., Hwang I.K., Won M.H., Rho J., Kwon Y.-G. (2005). TNF-Related Activation-Induced Cytokine Enhances Leukocyte Adhesiveness: Induction of ICAM-1 and VCAM-1 via TNF Receptor-Associated Factor and Protein Kinase C-Dependent NF-κB Activation in Endothelial Cells. J. Immunol..

[30] Machoń N.J., Zdanowska N., Klimek-Trojan P., Owczarczyk-Saczonek A. (2025). Vascular Cell Adhesion Molecule 1 and E-Selectin as Potential Cardiovascular Risk Biomarkers in Psoriasis. Int. J. Mol. Sci..

[31] Radić M., Belančić A., Đogaš H., Vučković M., Sener Y.Z., Sener S., Fajkić A., Radić J. (2025). Cardiometabolic Risk in Psoriatic Arthritis: A Hidden Burden of Inflammation and Metabolic Dysregulation. Metabolites.

[32] Morrone P., Caroppo F., De Pedrini A., Colletti A., Baj G. (2025). New Perspectives on Nutraceutical Insulin Sensitizing Agents in the Treatment of Psoriasis and Other Dermatological Diseases. Int. J. Mol. Sci..

[33] Li Q., Pang B., Dang E., Wang G. (2024). Endothelial Dysfunction in Psoriasis: An Integrative Review. J. Investig. Dermatol..

[34] Sieminska I., Pieniawska M., Grzywa T.M. (2024). The Immunology of Psoriasis—Current Concepts in Pathogenesis. Clin. Rev. Allergy Immunol..

[35] Armstrong A.W., Armstrong E.J., Fuller E.N., Sockolov M.E., Voyles S.V. (2011). Smoking and Pathogenesis of Psoriasis: A Review of Oxidative, Inflammatory and Genetic Mechanisms: Smoking and Pathogenesis of Psoriasis. Br. J. Dermatol..

[36] Shaharyar S., Warraich H., McEvoy J.W., Oni E., Ali S.S., Karim A., Jamal O., Blaha M.J., Blumenthal R.S., Fialkow J. (2014). Subclinical Cardiovascular Disease in Plaque Psoriasis: Association or Causal Link?. Atherosclerosis.

[37] Babalic F.C.A., Borza C., Ilie Rosca C., Gurban C.V., Banciu C.D., Mederle O.A., Popa M.D., Chelu S.C., Marius P., Sharma A. (2022). Endothelial Dysfunction in Psoriatic Arthritis Patients: Correlations between Insulin Resistance and Disease Activity. Eur. Rev. Med. Pharmacol. Sci..

[38] Gupta R., Shukla D., Saxena A., Prasad S. (2025). Correlation of Serum Cytokine Levels with Disease Severity in Psoriasis: A Physiological Insight into Inflammatory Markers. Bioinformation.

[39] Brezinski E., Follansbee M., Armstrong E., Armstrong A. (2014). Endothelial Dysfunction and the Effects of TNF Inhibitors on the Endothelium in Psoriasis and Psoriatic Arthritis: A Systematic Review. Curr. Pharm. Des..

[40] Knowles L., Nadeem N., Chowienczyk P.J. (2020). Do Anti-tumour Necrosis Factor-α Biologics Affect Subclinical Measures of Atherosclerosis and Arteriosclerosis? A Systematic Review. Br. J. Clin. Pharmacol..

[41] Eder L., Joshi A.A., Dey A.K., Cook R., Siegel E.L., Gladman D.D., Mehta N.N. (2018). Association of Tumor Necrosis Factor Inhibitor Treatment With Reduced Indices of Subclinical Atherosclerosis in Patients With Psoriatic Disease. Arthritis Rheumatol..

[42] Wegner J., Karbach S., Drosos I., Schnorbus B., Muxel S., Schmidt F., Wenzel P., Waisman A., Münzel T., Gori T. (2022). TNF-α Blockade May Lead to Improvement of Vascular Function in Psoriasis Patients. Exp. Dermatol..

[43] Lin T.-L., Fan Y.-H., Fan K.-S., Juan C.-K., Chen Y.-J., Wu C.-Y. (2025). Cardiovascular Disease Risk in Patients with Psoriasis Receiving Biologics Targeting TNF-α, IL-12/23, IL-17, and IL-23: A Population-Based Retrospective Cohort Study. PLoS Med..

[44] Nguyen T., Wu J.J. (2014). Relationship between Tumor Necrosis Factor-α Inhibitors and Cardiovascular Disease in Psoriasis: A Review. Perm. J..

[45] Sorour N., AbdulHafeez N., Akl E., Khater H., El-Shahat A. (2021). Assessment of Micro-Albuminuria and Renal Artery Resistance Index in Psoriatic Patients. Benha J. Appl. Sci..

[46] Di Caprio R., Nigro E., Di Brizzi E.V., Buononato D., Mallardo M., Tancredi V., Daniele A., Balato A. (2024). Exploring the Link Between Psoriasis and Adipose Tissue: One Amplifies the Other. Int. J. Mol. Sci..

[47] Gelfand J.M. (2025). The Visceral Relationship of Psoriasis and Obesity. J. Investig. Dermatol..

[48] Burhans M.S., Hagman D.K., Kuzma J.N., Schmidt K.A., Kratz M., Prakash Y.S. (2018). Contribution of Adipose Tissue Inflammation to the Development of Type 2 Diabetes Mellitus. Comprehensive Physiology.

[49] Kajikawa M., Higashi Y. (2022). Obesity and Endothelial Function. Biomedicines.

[50] Salihbegovic E., Hadzigrahic N., Suljagic E., Kurtalic N., Sadic S., Zejcirovic A., Mujacic A. (2015). Psoriasis and High Blood Pressure. Med. Arch..

[51] World Medical Association (2013). World Medical Association Declaration of Helsinki: Ethical Principles for Medical Research Involving Human Subjects. JAMA.

