# Supplementary material for: Endothelial Dysfunction in Psoriasis: A Comparative Analysis of Cutaneous, Inflammatory, and Metabolic Correlates
Source: Int J Mol Sci. 2026 Oct 29;27(21):9675. doi: 10.3390/ijms27219675 (PMC13762705; doi:10.3390/ijms27219675)
Supplement: Supplementary file 1 [file ijms-27-09675-s001.zip › ijms-4549185-supplementary.pdf]

Supplementary Table S1. Shapiro–Wilk test of normality for study variables

Variable	W	*p*-value	Distribution
PASI	0.897	<0.001	Non-normal
TNF-α	0.969	0.129	Normal
HOMA-IR	0.977	0.301	Normal
FMD	0.904	<0.001	Non-normal
RI	0.930	0.002	Non-normal

Variables with $p < 0.05$ were considered to deviate significantly from a normal distribution. PASI, Psoriasis Area and Severity Index; TNF-α, tumor necrosis factor alpha; HOMA-IR, homeostatic model assessment of insulin resistance; FMD, flow-mediated dilation; RI, renal resistive index.

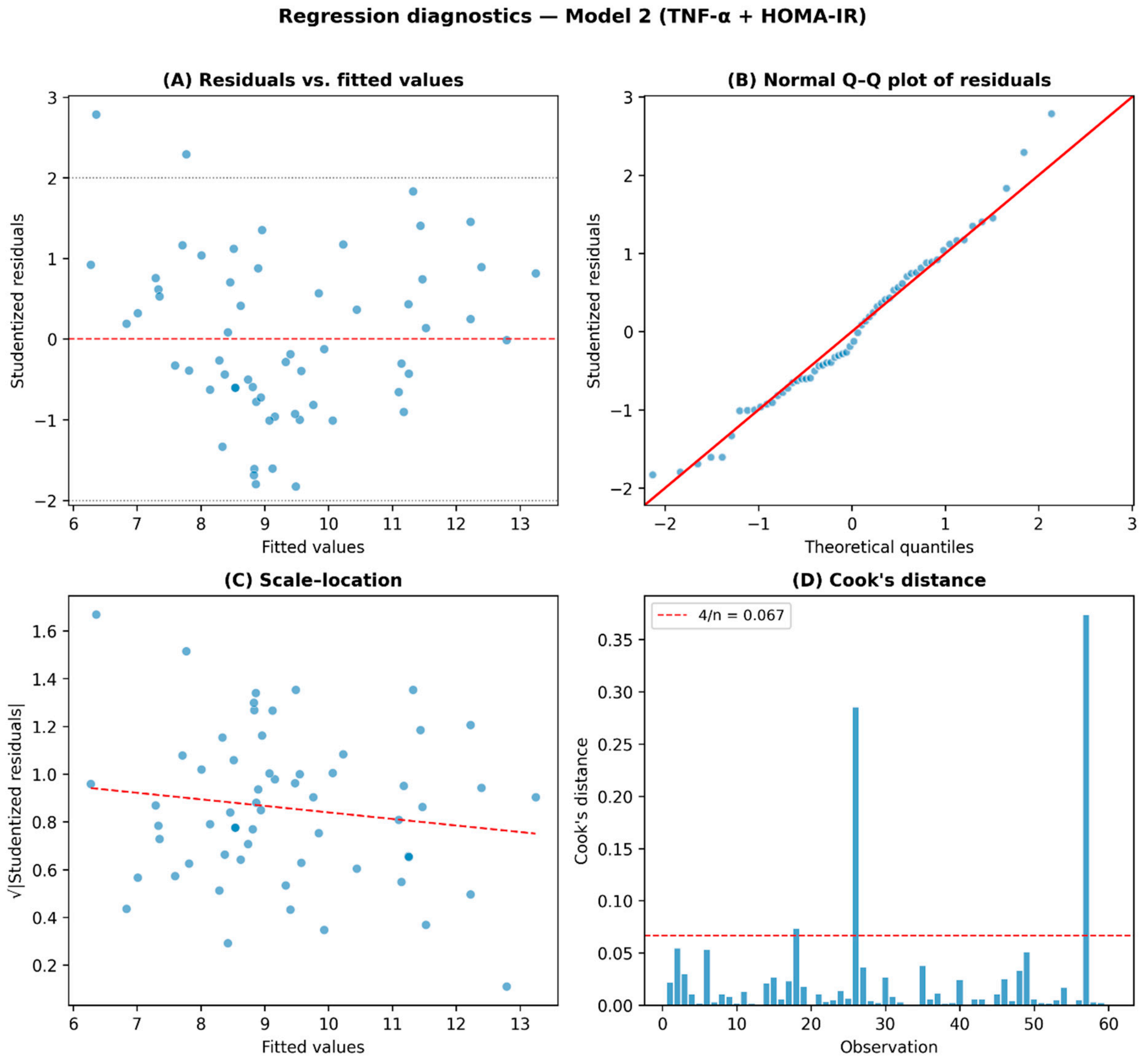

Supplementary Figure S1. Regression diagnostic plots for Model 2 (FMD ~ TNF-α + HOMA-IR). (A) Studentized residuals versus fitted values; residuals are randomly scattered around zero with no systematic pattern, supporting the assumptions of linearity and homoscedasticity (Breusch–Pagan test, $p = 0.051$). (B) Normal Q–Q plot of studentized residuals; points closely follow the theoretical diagonal, consistent with normally distributed residuals (Shapiro–Wilk $W = 0.983$, $p = 0.559$). (C) Scale–location plot (√|studentized residuals| versus fitted values); the near-flat trend line indicates constant variance

across the range of predicted FMD. (D) Cook's distance by observation number; the dashed line marks the 4/n threshold (0.067). Three observations exceeded this threshold (maximum Cook's D = 0.37), but none approached the conventional influence cutoff of 1.0, and excluding them did not materially alter the regression coefficients.

Supplementary Table S2. Multivariable linear regression models adjusted for confounders

Model	Predictor	β (unstd.)	95% CI	Std. β	p-value	VIF
**Base model (R^2 = 0.576, adj. R^2 = 0.537)**	Age	−0.010	[−0.052, 0.032]	−0.044	0.641	1.11
	Sex	0.050	[−0.695, 0.794]	0.013	0.894	1.20
	BMI	−0.031	[−0.230, 0.168]	−0.054	0.756	3.85
	SBP	−0.108	[−0.162, −0.054]	−0.664	<0.001	3.50
	Smoking	0.558	[−0.173, 1.288]	0.138	0.132	1.04
**Model B (R^2 = 0.740, adj. R^2 = 0.711)**	Age	−0.004	[−0.038, 0.029]	−0.018	0.805	1.11
	Sex	0.062	[−0.528, 0.651]	0.016	0.834	1.20
	BMI	0.022	[−0.136, 0.181]	0.039	0.778	3.91
	SBP	−0.053	[−0.100, −0.006]	−0.326	0.027	4.20
	Smoking	0.407	[−0.174, 0.987]	0.101	0.166	1.05
	HOMA-IR	−0.927	[−1.250, −0.605]	−0.596	<0.001	2.17
**Model C (R^2 = 0.676, adj. R^2 = 0.639)**	Age	0.004	[−0.034, 0.041]	0.016	0.853	1.15
	Sex	0.006	[−0.652, 0.664]	0.001	0.986	1.20
	BMI	0.074	[−0.110, 0.257]	0.129	0.424	4.19
	SBP	−0.094	[−0.143, −0.046]	−0.580	<0.001	3.57
	Smoking	0.546	[−0.099, 1.191]	0.136	0.095	1.04
	TNF-α	−0.056	[−0.084, −0.028]	−0.421	<0.001	1.78
**Model D (R^2 = 0.600, adj. R^2 = 0.554)**	Age	−0.002	[−0.044, 0.041]	−0.008	0.931	1.16
	Sex	0.002	[−0.731, 0.735]	0.001	0.996	1.20
	BMI	0.023	[−0.182, 0.229]	0.041	0.821	4.24
	SBP	−0.093	[−0.149, −0.037]	−0.572	0.002	3.57
	Smoking	0.511	[−0.208, 1.230]	0.127	0.160	1.05
	PASI	−0.045	[−0.096, 0.007]	−0.245	0.086	2.60

Base model includes age, sex, BMI, systolic blood pressure, and smoking status as prespecified covariates. Model B: base + HOMA-IR; Model C: base + TNF-α; Model D: base + PASI. β, regression coefficient; 95% CI, 95% confidence interval; Std. β, standardized regression coefficient; VIF, variance inflation factor; BMI, body mass index; SBP, systolic blood pressure; HOMA-IR, homeostatic model assessment of insulin resistance; TNF-α, tumor necrosis factor-alpha; PASI, Psoriasis Area and Severity Index.
